# Development of a High Irradiance LED Configuration for Small Field of View Motion Estimation of Fertilizer Particles

**DOI:** 10.3390/s151128627

**Published:** 2015-11-12

**Authors:** Simon Cool, Jan G. Pieters, Koen C. Mertens, Sergio Mora, Frédéric Cointault, Julien Dubois, Tim van de Gucht, Jürgen Vangeyte

**Affiliations:** 1 Institute of Agricultural and Fisheries Research, Burg. van Gansberghelaan 115, Merelbeke 9820, Belgium; E-Mails: Koen.Mertens@ilvo.vlaanderen.be (K.C.M.); Tim.Vandegucht@ilvo.vlaanderen.be (T.G.); Jurgen.Vangeyte@ilvo.vlaanderen.be (J.V.); 2 Department of Biosystems Engineering, Ghent University, Coupure links 653, Gent 9000, Belgium; E-Mails: Jan.Pieters@Ugent.be (J.G.P.); sermofo1@etsia.upv.es (S.M.); 3 UMR 1347 Agroecology INRA/Agrosup Dijon/University of Burgundy, 26 Bd Dr Petitjean, Dijon, 21000, France; E-Mail: frederic.cointault@agrosupdijon.fr; 4 Laboratoire Electronique, Informatique et Image, University of Burgundy, Allée Alain Savary 9, Dijon 21000, France; E-Mail: julien.dubois@u-bourgogne.fr

**Keywords:** LED illumination, genetic algorithm, fertilizer

## Abstract

Better characterization of the fertilizer spreading process, especially the fertilizer pattern distribution on the ground, requires an accurate measurement of individual particle properties and dynamics. Both 2D and 3D high speed imaging techniques have been developed for this purpose. To maximize the accuracy of the predictions, a specific illumination level is required. This paper describes the development of a high irradiance LED system for high speed motion estimation of fertilizer particles. A spectral sensitivity factor was used to select the optimal LED in relation to the used camera from a range of commercially available high power LEDs. A multiple objective genetic algorithm was used to find the optimal configuration of LEDs resulting in the most homogeneous irradiance in the target area. Simulations were carried out for different lenses and number of LEDs. The chosen configuration resulted in an average irradiance level of 452 W/m^2^ with coefficient of variation less than 2%. The algorithm proved superior and more flexible to other approaches reported in the literature and can be used for various other applications.

## 1. Introduction

In Europe, more than 90% of granular fertilizer spreaders are centrifugal spreaders [1,2], mainly because of their low cost and the large working width [3]. The spreading process is simple, but very difficult to control due to the complex interaction between fertilizer particles and the spreading equipment [4]. Working widths are continuously increasing, leading to an increased sensitivity to errors in the distribution of fertilizer particles. Spreader manufacturers provide spread charts for the user to find the optimal spreader settings for a given fertilizer and a chosen working width. Traditionally, these are determined by measuring the mass distribution of fertilizer on the ground. A totally different approach is to model the behavior of fertilizer particles and predict their landing positions based on a ballistic flight model. However, these landing positions cannot be predicted without knowing the velocity (typically 20–35 m/s) and direction of motion of individual particles. To obtain these parameters, different techniques were used in literature. Van Liedekerke *et al.* used DEM modeling to determine the velocities and the directions of the fertilizer particles but due to particle interactions, trajectories on the disk were very difficult to model [5]. Grift and Hofstee developed an optical system based on photosensitive sensors to determine the horizontal velocity and diameter of particles leaving the spreading disks [6]. The main drawback is that only the radial component of the velocity vectors can be determined. More recent approaches are inspired by Particle Image Velocimetry (PIV), where tracer particles are seeded in a fluid to predict global velocity fields of flows based on motion of individual particles [7]. Similarly they use image acquisition and processing to determine two or three dimensional information about the moving particles. Cointault *et al.* proposed a multi exposure image acquisition system using a series of flashes and a field of view of 1 m × 1 m allowed capturing one throw of fertilizer 8 times in one image [8]. By subsequently switching the light on and off while the camera shutter is open, particles are captured multiple times on the same image. Cointault and Vangeyte combined a high resolution monochrome camera with a stroboscope system [9]. They used a small field of view of 0.1 m × 0.1 m and a LED stroboscope to create multi-exposure images. Hijazi *et al.* developed a two-step cross-correlation–based algorithm for motion estimation of the fertilizers particles and validated it with simulated images [10]. In the set-up of [8,9], only one camera was used, and no depth information could be obtained without prior knowledge of the disk configuration and position. Even with flat disks, fertilizer particles are not ejected in a common plane [11]. In practice, conical disks are often used to give the fertilizer particles an upward velocity component to increase the spreading distance and subsequently the working width. Villette *et al.* determined the horizontal outlet angle of particles leaving the spreading disks using motion blurred images and derived the three dimensional velocity vectors using a mechanical model [11]. The vertical component of the velocity vector was estimated very accurately. However from their images, it is not possible to estimate particle diameters, which is a crucial parameter when predicting landing positions [12]. Moreover, application is limited to spreading disks with straight vanes assuming that particles slide along the disk. Hijazi *et al.* developed a high speed stereo image acquisition system to counter these problems [13]. As in the studies of [11,14], a field of view of 1 m × 1 m was used to capture full throws of fertilizer in one image. The pixel to mm ratio was very small (about 1 pixel/mm), introducing a high sensitivity for errors in the 3D positioning. Furthermore, this hampers an accurate estimation of the particle diameters. These shortcomings can be solved using a smaller field of view (0.3 m × 0.3 m). Although this reduces the amount of particles that are visible in the images, it increases the particle resolution which in its turn increases the accuracy of the 3D positioning. Moreover, it allows for a more accurate estimation of the particle diameters and even of the particle shape. In previous studies [12,15,16], it has been shown that the particle shape, influencing the aerodynamic drag coefficient, has a major effect on the traveled distance of the particles, and confirms its great impact when predicting landing positions of fertilizer particles.

Since a small exposure time is necessary to reduce motion blur of moving particles and a relative large F-number is used to maximize the depth of field, sufficient illumination is necessary to ensure that particles are sufficiently visible on the images to segment them from the background and noise. Furthermore, the light should be distributed homogeneously in the field of view to prevent under-segmentation of the images. A qualitative segmentation allows an accurate estimation of the particle diameter and an improved accuracy of the 3D motion estimation. No custom-of-the-shelf commercial system is available delivering this large amount of light at high uniformity. Therefore, this paper deals with the development of a lighting system to provide these specific requirements. In future work, the developed lighting system will be used in combination with a stereovision setup and motion estimation algorithms (see [13]) to determine accurately the spread pattern of centrifugal fertilizer spreaders.

## 2. Theoretical Background

LEDs have a number of advantages over traditional lighting sources and were therefore selected for this application. They have a relatively low energy consumption, long lifetime and small size, and moreover, various colors are available [17]. Furthermore, they allow fast switching, which is important when synchronizing the lighting system with a high speed camera. LEDs are often combined in arrays to achieving a suitable light intensity and homogeneity since a single LED cannot provide sufficient illumination, and because of their lighting distribution pattern. The overall illumination pattern of an array of LEDs is obtained by superposition of the different illumination patterns of each LED [18]. There are different methods for designing LED arrays for obtaining high uniform irradiance patterns. Moreno *et al.* proposed a method to determine the optimum packaging density of square, linear, circular and hexagonal LED arrays for imperfect Lambertian sources by determining the optimum LED to LED spacing for a given distance between LED source and target [19]. Yang *et al.* showed the uniformity as a function of this distance [19]. They presented two general results: the first one states the scaling property of the basic illumination pattern (illumination pattern of a single LED), the second claims that for any basic illumination pattern and grid shape for the LED array, the radiation pattern with maximum uniformity can be always achieved by setting the luminous intensity levels of all the LEDs to be identical.

Some studies focus on the design of optical components to achieve uniform illumination from LED sources, e.g., [20] designed a method to optimize the light intensity distribution curve (LIDC), achieving highly uniform illumination (when distance-height ratio (DHR) is given) by designing freeform lenses. Whang *et al.* [21] and Cheng and Nong [22] proposed solutions for uniform lighting with three kinds of illumination systems: circular ring arrays, linear arrays and square arrays. All the methods presented above are analytical methods, based on Sparrow’s Criterion [19]. This means that the illumination uniformity is only assessed over the central region of the target plane. For general applications, standard configurations have been proposed [23].

For applications where uniformity is required over the whole target plane, as is the case in our situation, Lei *et al.* developed a local search algorithm to obtain a highly uniform illumination, considering the whole target plane [17]. An initial random condition is iteratively improved by moving LEDs to neighboring positions of candidate solutions. Their algorithm minimizes an objective function, being the Coefficient of Variation of the illuminance distribution in a plane perpendicular to the LED array, to obtain the highest uniformity. The algorithm does not control the illuminance level, is very sensitive to local optima and should therefore be calculated with a considerable number of initial random positions.

## 3. Material and Methods

### 3.1. General Requirements

The aim of this paper is to create a high irradiance lighting system which will be used in future experiments for motion estimation of fertilizer particles using stereovision. Image acquisition is done in a controlled environment to exclude illumination variability and to promote repeatability during experiments. Based on preliminary fertilizer spreading experiments, it was determined that a minimum irradiance of 450 W/m^2^ will be necessary. The irradiance homogeneity should be maximized as much as possible and a threshold of 2% was chosen. An algorithm will be used to find the optimal LED configuration to deliver a high irradiance level to a target area of 0.3 on 0.3 m, which is the field of view of the cameras. The distance from the lighting source to the target plane was set equal to that of the cameras (0.6 m). For practical purposes, the size of the LED array was limited to 0.5 m × 0.5 m. In the center of the LED array, space needs to be provided for the lenses of the cameras, which are placed 150 mm apart. To determine the optimal number of LEDs and the individual LED positions, a multiple objective genetic algorithm was used.

### 3.2. Lighting Calculations

Generally, there are two ways to quantify optical radiation. Radiometry is the measurement of electromagnetic radiation within the frequency range 0.3 and 3000 THz, while photometry is restricted to the frequencies detectable by the human eye. General photometric and radiometric quantities and units are illustrated in Table 1.

**Table 1:** General photometric and radiometric quantities.

Quantity	Photometric	Radiometric
Energy per unit time	Luminous flux (lm)	Radiant flux (W)
Power per unit area	Illuminance (lx)	Irradiance (W/m^2^)
Power per unit solid angle	Luminous intensityn (cd)	Radiant intensity (W/sr)
Power per area solid angle	Luminance (cd/m^2^)	Radiance (W/m^2^/sr)

Most authors assume that LEDs have Lambertian distribution patterns, meaning that the luminous intensity I is a cosine function of the viewing angle, being the angle off-center (0°) [19]:

(1) $$\text{I}\left(\text{θ}\right)={\text{I}}_{0}{\text{ cos}}^{\text{m }}\text{θ}$$

with I0 being the luminous intensity at the normal direction to the source surface (Cd) and θ the viewing angle (°).

The parameter m is calculated using the half width viewing angle θ12 which is the viewing angle at which the radiant intensity is half of the value at the normal direction. θ12 is calculated as half of the Full Width at Half Maximum value (FWHM) and is generally provided by the manufacturer. This parameter is calculated according to:

(2) m=−ln2ln( cosθ12 )

**Figure 1:**
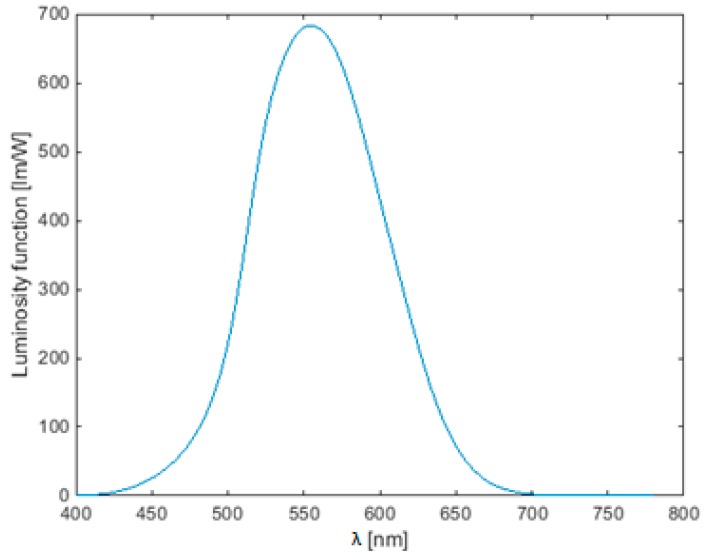
Standard luminosity function for photopic vision.

In some cases, the manufacturer provides the luminous intensity distribution for a LED-lens combination. This was the case for the application in this paper. This photometric data was first converted to radiometric units by weighting with the standard luminosity function (see Figure 1). The best fitting Gaussian function (order n) was used for interpolation of the irradiance values:

(3) $$\text{I}\left(\text{θ}\right)=\overset{n}{\underset{i=1}{\sum }}a_{i}{\text{e}}^{-{\left(\frac{\theta -b_{i}}{c_{i}}\right)}^{2}}$$

where I0 is the radiant intensity in the normal direction to the source surface (W/sr), and $a_{i},\;b_{i}\text{and }c_{i}$ are constant values depending on the function fit.

The radiant to luminous flux conversion factor ${\text{η}}_{v}$ is defined as the ratio of the luminous flux (given by the manufacturer) to the radiant flux of the LED. Because the individual LEDs can have different positions with respect to the lighting source and can also be oriented in different ways, three coordinate systems can be defined. The first coordinate system is the LED coordinate system; the second is the source coordinate system, situated in the geometric center of the LED array. The third coordinate system is the target coordinate system (x, y, z), situated in the geometric center of the target that needs to be illuminated.

The irradiance E (W/m^2^) at point i, illuminated by LED j, can be calculated using the “cosine Law” or “inverse square law”:

(4) $${\text{E}}_{\text{i},\text{j}}=\frac{\text{I}\left({\text{θ}}_{\text{i},\text{j}}\right)\cos{\text{θ}}_{\text{i},\text{j}}}{{\text{r}}_{\text{i},\text{j}}^{2}}$$

with ${\text{r}}_{\text{i},\text{j}}$ being the distance between the point i and the LED j.

Let $P_{\text{T}}=\left[\begin{array}{c} {\text{x}}_{\text{i}}\\ {\text{y}}_{\text{i}}\\ {\text{z}}_{\text{i}}\\ 1 \end{array}\right]$ be the position of point i in the target coordinate system (homogeneous coordinates), the position in the coordinate system of LED j can be calculated as:

(5) $$\left[\begin{array}{c} {\text{a}}_{\text{i},\text{j}}\\ {\text{b}}_{\text{i},\text{j}}\\ {\text{c}}_{\text{i},\text{j}}\\ 1 \end{array}\right]=\left[\begin{array}{cc} R & T\\ 0 & 1 \end{array}\right]P_{\text{T}}$$

with:

(6) $$\left[\begin{array}{cc} R & T\\ 0 & 1 \end{array}\right]=H_{\text{S}\to \text{L}}\;H_{\text{T}\to \text{S}}$$

where $H_{\text{T}\to \text{S}}$ is the transformation matrix from the target to the source coordinate system and $H_{\text{S}\to \text{L}}$ the transformation matrix from the source to the LED coordinate system.

Since $\cos{\text{θ}}_{\text{i},\text{j}}=\frac{{\text{c}}_{\text{i},\text{j}}}{{\text{r}}_{\text{i},\text{j}}}$ and from Equation (4), we can find the irradiance as a function of the position in the target coordinate system:

(7) $${\text{E}}_{\text{i},\text{j}}=\frac{\text{I}({\text{θ}}_{\text{i},\text{j}})([R_{3}\;{\text{T}}_{3}]P_{T})}{{\left({\left([R_{1}{\text{T}}_{1}]P_{T}\right)}^{2}{\left([R_{2}{\text{T}}_{2}]P_{T}\right)}^{2}{\left([R_{3}{\text{T}}_{3}]P_{T}\right)}^{2}\right)}^{\frac{3}{2}}}$$

where the subscript for R and T indicates the row of the matrix.

In the case that LEDs are positioned horizontally on a source plane and the source plane is horizontally aligned with the target, $\left[\begin{array}{cc} R & T\\ 0 & 1 \end{array}\right]$ becomes $\left[\begin{array}{cccc} 1 & 0 & 0 & -{\text{u}}_{\text{j}}\\ 0 & 1 & 0 & -{\text{v}}_{\text{j}}\\ 0 & 0 & 1 & {\text{w}}_{\text{i}}\\ 0 & 0 & 0 & 1 \end{array}\right]$ with $({\text{u}}_{j}$, ${\text{v}}_{j})$ the position of LED j in the source coordinate system (2D) and ${\text{w}}_{\text{i}}$ the vertical distance of point i to the source plane.

In [24] a simple equation to evaluate the far-field condition of a LED array in function of the LED radiation pattern, array geometry and number of LEDs was developed. Generally, the “rule of five” can be used, stating that a LED array can be modelled as a directional point source if $\frac{r_{min}}{D}$ is larger than five, with $r_{min}$ the minimal distance from source to target (resulting in an error of less than 1%) and D the largest dimension of the source array. When this is not the case, for example because of the considerable size of the LED array, each LED needs to be modeled separately.

When n LEDs are used, the total irradiance can be calculated as:

(8) $${\text{E}}_{\text{i}}=\overset{\text{n}}{\underset{\text{j}=1}{\sum }}{\text{E}}_{\text{i},\text{j}}$$

Illumination uniformity can be assessed in different ways [24]. Amongst these parameters, the coefficient of variation is most widely used:

(9) $$\text{CV}=\frac{{\text{σ}}_{\text{E}}}{\bar{\text{E}}}$$

with $\bar{E}$ and ${\text{σ}}_{E}$ being the irradiance average and standard deviation in the target plane, respectively.

### 3.3. Multiple Objective Genetic Algorithm

Most studies in the literature seek to optimize the illuminance uniformity. The illuminance level is generally considered in these approaches and is set using the number of LEDs or the size of the source plane. The purpose of this study is to find the optimal configuration of LEDs delivering high irradiance in the target area. High intensity is necessary to maximize the depth of field and minimize motion blur. However, uniformity is also important to reduce under-segmentation and increase the accuracy of the matching steps [13]. To deal with these two optimization problems, a multiple objective optimization was used.

When the target area is discretized to a high level, the search space for the algorithm is very large. Especially when a high number of LEDs is used, the problem becomes very complex. Due to their broad range and efficiency [25], a genetic algorithm was chosen for this application. This family of algorithms uses the evolution of a population over subsequent generations to search the optimal solution for a target problem. A population is defined as a set of possible solutions, *i.e.*, individuals. Offspring is generated in two ways. Cross-over combines two parents to create a new child, while mutation introduces small random changes in the individuals creating offspring. Parents for the next generation are selected based on their fitness, which is their objective function value. Individuals with a better objective function value are given more chances to reproduce. Populations can be divided into subpopulations and migration between these subpopulations can be set. Eventually, the best individual resulting from the evolution process is chosen as the optimal solution [26]. The algorithm was implemented using the Global Optimization Toolbox of Matlab 2014b (Mathworks Inc., Natick, MA, USA).

The aim in this paper is to find the optimal position for each LED to maximize the average irradiance (radiometric units) and homogeneity of light distribution in the target plane. Therefore two objective functions were used. In most cases where multiple objectives are considered, there is no single solution that simultaneously optimizes both objective functions. Thus multiple Pareto-optimal solutions can be found. From this set of solutions, the most preferred solution can be selected, representing the optimal solution of the multiple objective problem.

**Figure 2:**
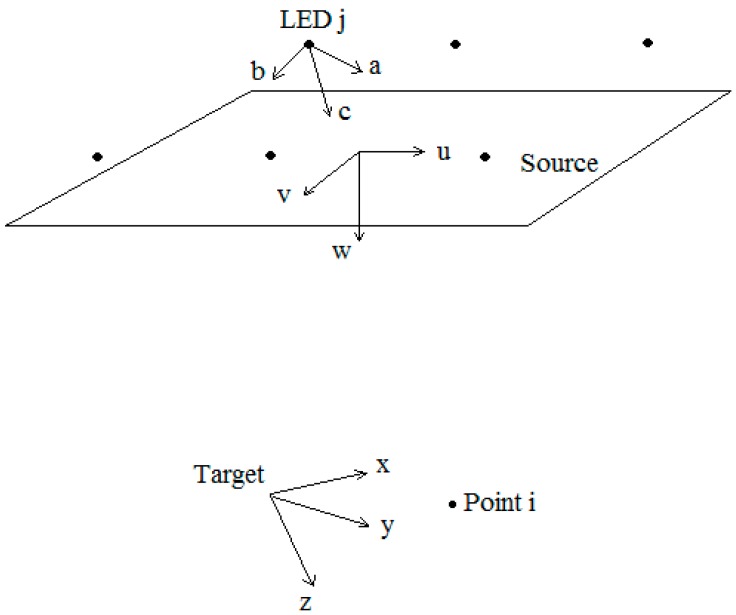
LED, source and target coordinate system.

In this study, the algorithm variables for which the objective functions needed to be optimized were the discrete x, y positions of the LEDs. As mentioned before, the source is reduced to a plane which is horizontally aligned with the target plane. This reduces the size of the search space by a large extent. The origin of the source plane is situated at the same distance from the target plane as the camera center at the height of the lens: (0 m, 0 m, 0.6 m) in the source coordinate system (see Figure 2). Therefore there are only two degrees of freedom for each LED: the discrete x- and y-position. To enforce symmetry into the system and reduce the number of degrees of freedom even more, the search space is reduced to one quadrant. The LEDs in the second, third and fourth quadrants take their x, y position accordingly. This restricts the number of LEDs used to be a multiple of four. Furthermore, a central LED will not be approved by the algorithm. Therefore, a configuration k with n different LEDs can be represented by a matrix ${\text{P}}_{\text{k}}$:

(10) $${\text{P}}_{\text{k}}=\left[x_{k},y_{k}\right]$$

with: $x_{k}$ and $y_{k}$ row vectors containing the x- and y-positions of the LEDs in the first quadrant.

The objective functions that must be optimized represent the radiant intensity, which should be maximized and the homogeneity, represented by the CV which should be minimized. Two-step linear objective functions were used in this study to make sure that solutions below the desired thresholds (of both irradiance and CV) were penalized to a higher degree than solutions above the thresholds were favored:

(11) $${\text{f}}_{1}\left(\bar{\text{E}}\right)=\{\begin{array}{c} {\text{a}}_{1}\left({\bar{\text{E}}}_{k}-\hat{\text{E}}\right)+{\text{b}}_{1},\;{\bar{\text{E}}}_{\text{k}}<\hat{\text{E}}\\ {\text{c}}_{1}\left({\bar{\text{E}}}_{\text{k}}-\hat{\text{E}}\right)+{\text{d}}_{1},\;{\bar{\text{E}}}_{k}\geq \hat{\text{E}} \end{array}$$

with: $\hat{\text{E}}$ the target irradiance = 450 W/m2. ${\bar{\text{E}}}_{k}$ is the average irradiance in the target area reached by configuration k.

Irradiance levels below the target irradiance level are inferior while irradiance levels above this value are superior, although to a lower degree: ${\text{a}}_{1}$ < ${\text{c}}_{1}$. The objective function for the CV is similar:

(12) $${\text{f}}_{2}\left(\text{CV}\right)=\{\begin{array}{c} {\text{a}}_{2}\left({\text{CV}}_{\text{k}}-\hat{\text{CV}}\right)+{\text{b}}_{2},\;{\text{CV}}_{\text{k}}<\hat{\text{CV}}\\ {\text{c}}_{2}\left({\text{CV}}_{k}-\hat{\text{CV}}\right)+{\text{d}}_{2},\;{\text{CV}}_{\text{k}}\geq \hat{\text{CV}} \end{array}$$

with: $\hat{\text{CV}}$ the target coefficient of variation = 2%.

**Figure 3:**
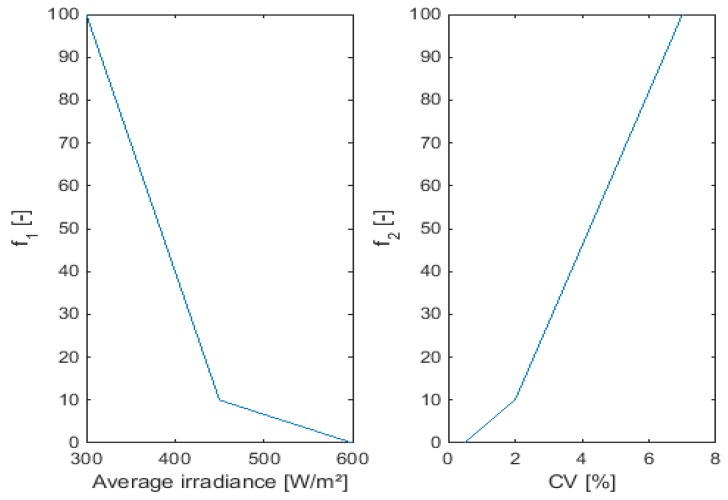
Objective functions used for the multiple objective genetic algorithm. (**Left**): average irradiance; (**Right**): Coefficient of Variation (CV).

Figure 3 illustrates the objective functions used for the simulations.

Inherent algorithm variables such as population size (150) and recombination to mutation ratio (4), but also the number of generations (150) were selected based on exploratory simulations (results not presented here). No subpopulations were used in the simulations.

A physical limit was set on the x, y positions to set a maximum on the array size. Furthermore, because we know there is a relation between the size of the source plane and the average irradiance level in the target area, this limit ensures that no Pareto optimal solutions are found with very large uniformity but low intensity. Another restriction implemented was to prevent the LEDs from overlapping and to ensure LED spacing for thermal management.

### 3.4. Simulations

#### 3.4.1. LED Selection

As a first step, the optimal LED was selected from a range of high power LEDs based on the spectral sensitivity of the camera. The following efficiency factor was calculated:

(13) $${\text{η}}_{\text{sens}}=\;\int_{{\text{λ}}_{\text{min}}}^{{\text{λ}}_{\text{max}}}{\text{ P}}_{\text{led}}\left(\text{ λ}\right){\text{ S}}_{\text{camera}}\left(\text{λ}\right)\partial \text{λ}$$

with: ${\text{P}}_{\text{led}}$ the normalized (area under the curve equals 1) radiant power of the LED as a function of the wavelength λ (W) and ${\text{ S}}_{\text{camera}}$ the relative spectral sensitivity function of the camera (–).

By multiplying ${\text{η}}_{\text{sens}}$ with the radiant flux of the LED (W), the camera spectral sensitivity factor (%) can be calculated:

(14) $${\text{F}}_{\text{camera}}={\text{ P}}_{\text{led}}\;{\text{η}}_{\text{sens}}\;100$$

This factor represents the radiant power of the LED as perceived by the camera relative to the total radiant power emitted by the LED.

The LEDs considered are given in Table 2.

**Table 2:** The luminous flux ${\text{ϕ}}_{v}$ and the wavelength at peak intensity ${\text{λ}}_{\text{peak}}$ are given. All values were provided by the manufacturer (Phillips Lumileds, San Jose, CA, USA).

LED Number	${\text{ϕ}}_{v}$ (lm)	$\lambda_{peak}$ (nm)	Name	Part Number
1	313	567.5	Lime	LXML-PX02-0000
2	122	505.0	Cyan	LXML-PE01-0070
3	161	530.0	Green	LXML-PM01-0100
4	320	-	Cool white	LXML-PWC2
5	310	-	Neutral white	LXML-PWN2
6	21	447.5	Royal blue	LXML-PR01
7	106	627.0	Red	LXM2-PD01-0050
8	140	590.0	Amber	LXML-PL01

#### 3.4.2. LED Configuration

Next, the optimal LED configuration was determined, minimizing the CV in the target area. The latter is defined as the field of view of the camera, which is approximately 0.30 × 0.30 m at 0.6 m distance in the z-direction. To focus the light to the target, three types of lenses were used, as given in Table 3.

**Table 3:** Lens specifications used for simulation (Carclo Optics, Ayesbury, UK).

Lens	Cdlm Value (Cd/lm)	FWHM (°)	Diameter (mm)	Part Number
Narrow	4.60	23.0	23	10611
Medium	2.59	28.64	23	10612
Wide	1.29	44.4	23	10613

In total, 36 LEDs were used in combination with the lenses above. First of all, two traditional approaches were used: a square and circular array of LEDs was simulated for different sizes of the source plane. The empirical formula of [19] was used to determine the optimal LED spacing in case of a square or circular array of LEDs. The multiple objective genetic algorithm was used as well to determine the optimal LED configuration and results were compared. Simulations were done for a different number of LEDs.

## 4. Results and Discussion

### 4.1. LED Selection

The efficiency factor ${\text{η}}_{\text{sens}}$ and the camera spectral sensitivity factor ${\text{F}}_{\text{camera}}$ were calculated for the different LEDs to select the most appropriate LED for this application. The results are given in Table 4.

**Figure 4:**
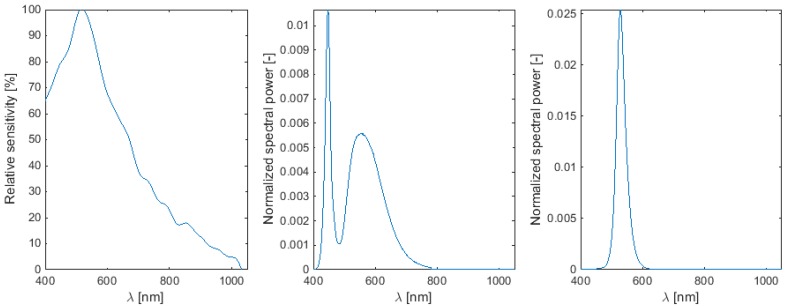
(**Left**): Relative camera sensitivity; (**Middle**): Normalized spectral power distribution for LED 4; (**Right**): Normalized spectral power distribution for LED 3.

The selection of the LED was only based on radiant efficiency, price was similar and was not considered here. From Table 2, it can be seen that LED 4 and LED 5 had the largest radiant flux (also with n°6 and n°7). However, this does not imply that the LED will result in a higher brightness on the images of the camera sensor, because the sensitivity of the sensor needs to be considered as well. Figure 4 shows the camera sensitivity and the normalized spectral power distribution for LEDs 3 and 4. From Table 4, it is clear that the efficiency factor is largest for LED 3, because the LED had a spectral power distribution very near the maximal sensitivity value of the camera. This is illustrated in Figure 4. This was not the case for LEDs 4 and 5. However, when considering the camera spectral sensitivity factor, taking into accounts both the camera sensitivity and the LED radiant flux, the highest value was obtained for LED 4. The higher radiant flux overrules the lower camera spectral sensitivity for this LED.

**Table 4:** Radiant to luminous flux conversion factor ${\text{η}}_{V}$, radiant flux φ (at given forward current $I_{f}$), camera spectral efficiency factor ${\text{η}}_{\text{sens}}$ and camera spectral sensitivity factor ${\text{F}}_{\text{camera}}$ for different LEDs.

LED Number	$I_{f}$ (mA)	$\eta_{v}$ (lm/W)	φ (W)	${\text{η}}_{sens}$ (%)	$F_{camera}$ (W)
1	700	461.4	0.678	80.5	54.6
2	700	213.4	0.572	92.0	52.6
3	700	551.9	0.292	95.6	27.9
4	1000	329.6	0.971	78.1	75.8
5	1000	348.4	0.890	74.6	68.5
6	700	20.0	0.910	76.5	69.6
7	700	97.0	1.093	56.1	61.3
8	700	472.3	0.296	70.2	20.8

### 4.2. LED Configuration

#### 4.2.1. Square and Circular Array

**Figure 5:**
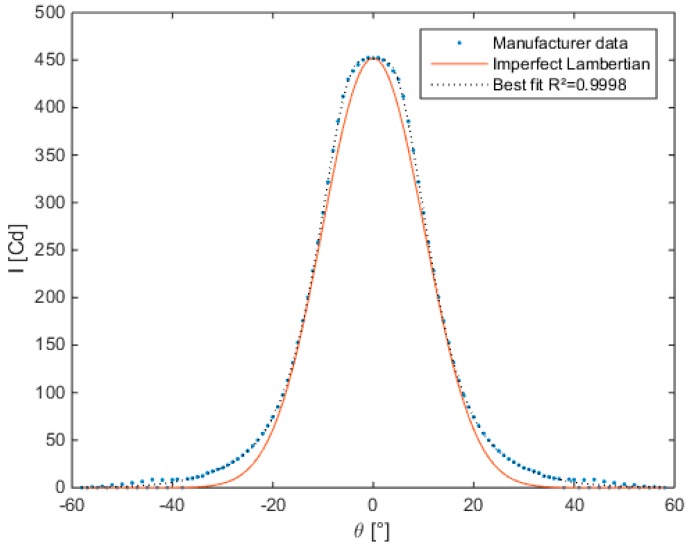
Luminous intensity distribution for narrow angle lens combined with the selected LED (data obtained at forward current of 350 mA, luminous flux of 100 lm).

**Figure 6:**
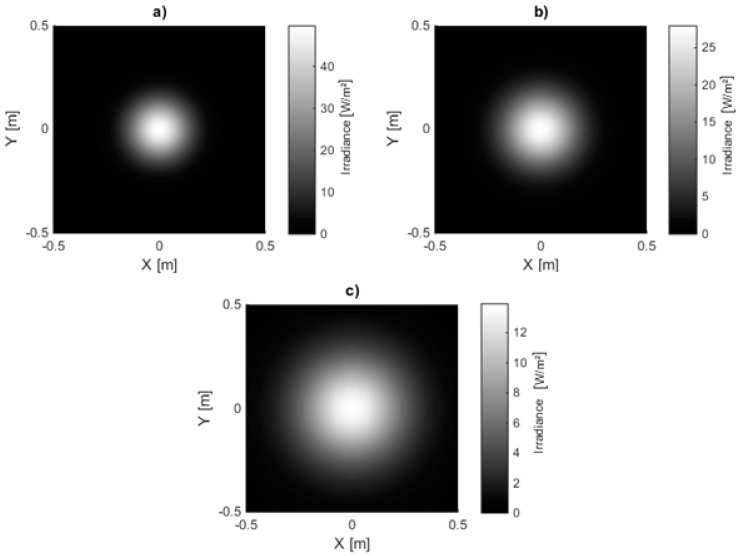
Simulated light distribution pattern at 0.6 m distance from LED 4 for (**a**) Narrow; (**b**) Medium; (**c**) Wide angle lens.

The angular luminous intensity distribution was provided by the manufacturer for the three types of lenses. Figure 5 illustrates the values for the narrow angle lens. The best fitting Gaussian function and the imperfect Lambertian distribution which is based on the half-angle are illustrated as well. We can see that the Lambertian distribution is not the ideal fit. In Figure 6, the irradiance distribution pattern for the LED in combination with the three types of lenses simulated for a plane with size 1 m at 0.6 m distance is given.

An array of LEDs can be considered as a point source if the size of the source is small relative to the distance from source to target. Generally, the “rule of 5” applies for the ratio of this distance and the largest source dimension (${\text{r}}_{\text{min}}$/D). This would result in an array with maximal dimension of 0.12 m, which is actually almost the smallest possible 6 × 6 LED array since the LEDs have a diameter of 23 mm. Moreno found for Lambertian emitters that the ${\text{r}}_{\text{min}}$/D was dependent on the number of LEDs used and the FWHM value [24]. For Lambertian emitters, values up to 70 were necessary for the far field condition to be satisfied. This illustrates that the light source in this paper could be considered as a point source and light distribution should be modelled for each LED separately.

**Figure 7:**
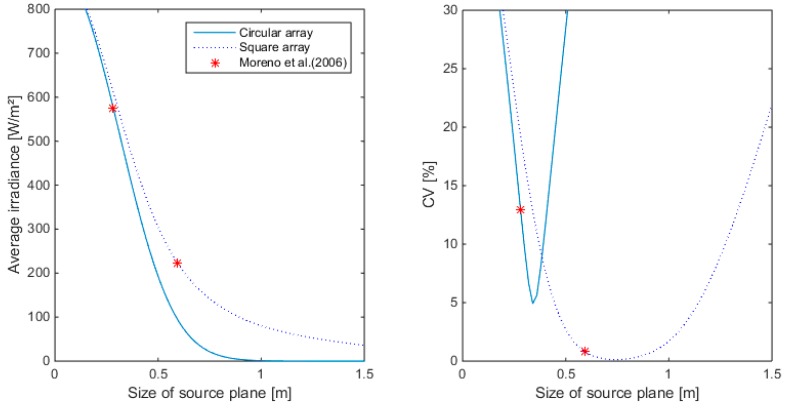
Average irradiance and Coefficient of Variation (CV) as a function of the source plane size for square and circular arrays of 36 LEDs. Simulations are done with the narrow beam lens. (**Left**): Average irradiance; (**Right**): CV.

**Figure 8:**
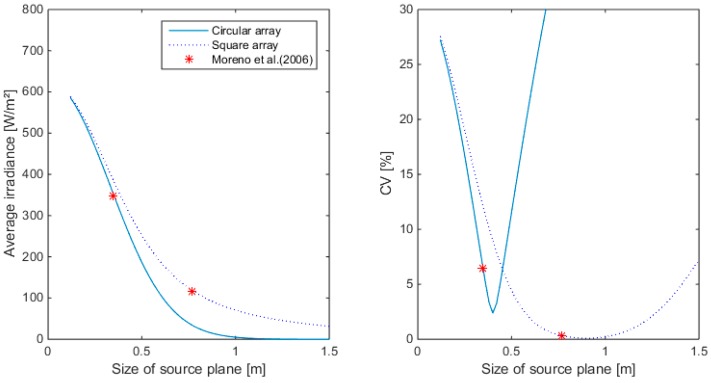
Average irradiance and CV as a function of the source plane size for square and circular arrays of 36 LEDs. Simulations are done with the medium beam lens. (**Left**): Average irradiance; (**Right**): CV.

**Figure 9:**
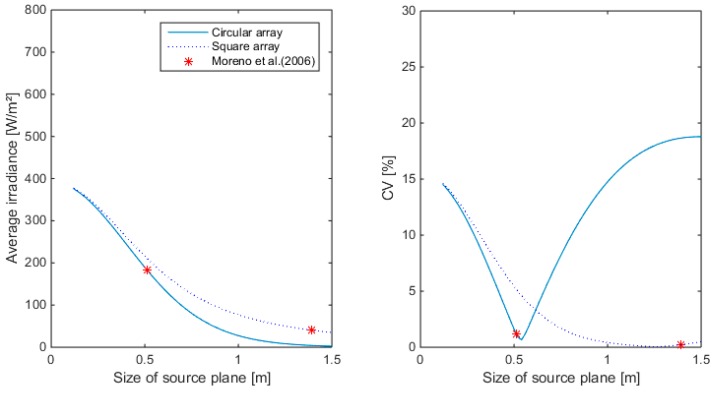
Average irradiance and CV as a function of the source plane size for square and circular arrays of 36 LEDs. Simulations are done with the wide beam lens. (**Left**): Average irradiance; (**Right**): CV.

Two different popular configurations were simulated: the square and the circular array. Results for 36 LEDs are illustrated in Figure 7, Figure 8 and Figure 9 for different sizes of the source plane for narrow, medium and wide angle lenses, respectively. The size of the source plane had a large effect on the average irradiance and CV in the target area. When the source plane increased in size, the average irradiance decreased. For both the square and circular array of LEDs, the CV decreases with the size of the source plane, then reaches a minimum and then increases again. For the square array with narrow lenses, the smallest CV value was found at an array size of 0.76 m. Although the CV here was very small (0.76%), the irradiance was too low for this application, since an irradiance of 450 W/m^2^ was required. The same was found for the medium and wide angle lens. Higher intensity values were found for a smaller source plane size, however, due to the suboptimal overlap between individual LED light distribution patterns, this resulted in a lower uniformity. With the wide angle lens, it is not possible to obtain more than 400 W/m^2^, not even with the smallest configuration. The lowest CV for the circular array was 5% at an irradiance level of 463.8 W/m^2^ for the narrow lens. Although the intensity was above the required level, the CV was too high, indicating a low uniformity level. For the medium and wide angle lenses, the intensity at the source plane size with lowest CV was lower than the required level. Generally, it can be seen that in case of a circular array, the intensity drops down faster with increasing source plane size compared to the square array for the same number of LEDs. On Figure 9, it can also be noticed that the CV as calculated by the empirical formula of [19] is not the minimum CV for a square and circular array. This can be attributed to the fact that the central region which is illuminated uniformly is different from the target area considered in this paper. For 36 LEDs, the narrow angle lens seems to be the only one appropriate for this application.

#### 4.2.2. Multiple Objective Genetic Algorithm

**Figure 10:**
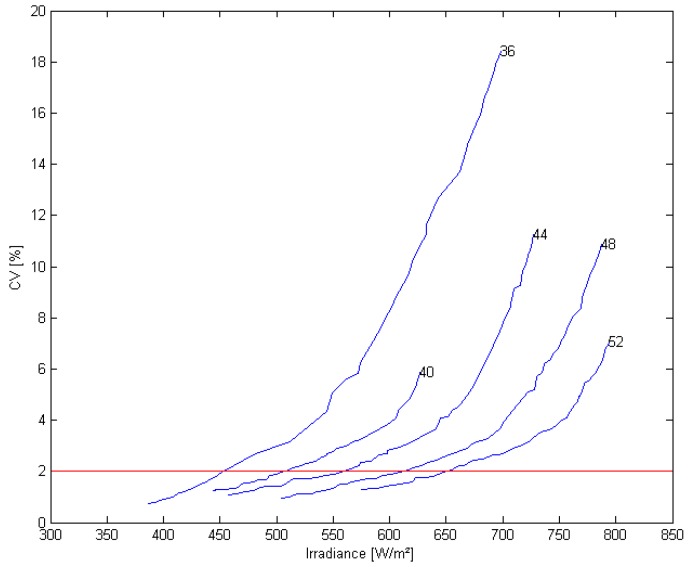
Pareto front for 36, 40, 44, 48 and 52 LEDs. The CV limit of 2% is given by the horizontal line.

For the narrow lens, simulations with 36 to 52 LEDs were made in steps of four. Simulations with less LEDs resulted in an insufficient irradiance level and were excluded. A minimum spacing of 25 mm between LED centers was used for thermal reasons. Results are given in Figure 10.

From Figure 10, we can see that the higher the number of LEDs, the more the Pareto front moves to higher irradiance levels. Placing the upper limit for the CV at 2%, the following values were found (Table 5).

**Table 5:** Average irradiance at CV = 2% for different number of LEDs.

Number of LEDs	Average Irradiance (W/m^2^)	CV (%)
36	452	1.98
40	504	1.97
44	558	1.99
48	616	2.05
52	648	1.95

**Figure 11:**
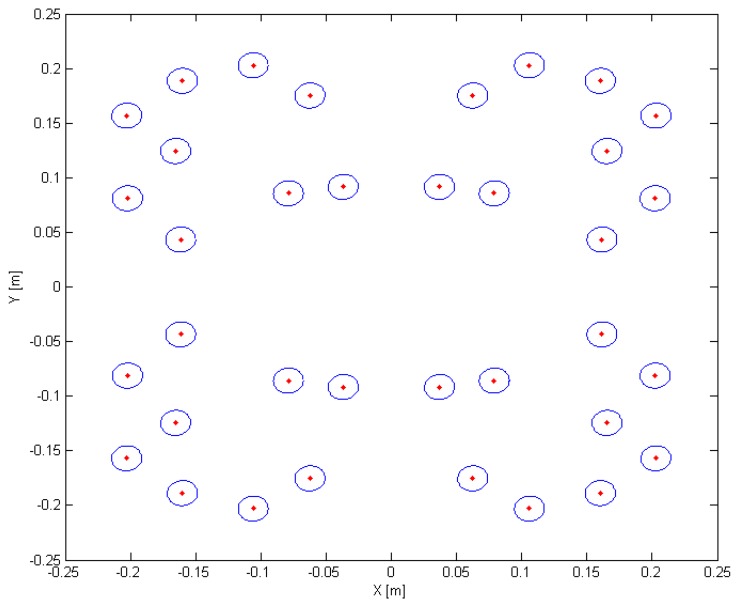
Optimal LED positions calculated with the multiple objective genetics algorithm for a number of 36 LEDs. The search space was reduced to a plane at 60 cm from the target plane and all LEDs are oriented parallel with this plane.

**Figure 12:**
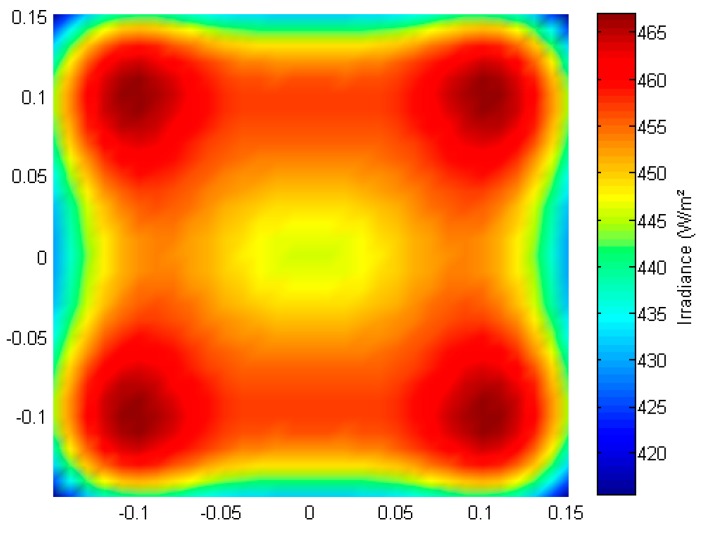
Irradiance distribution for optimal configuration obtained with a multiple objective genetic algorithm for a number of 36 LEDs.

The minimal irradiance level was set at 450 W/m^2^ and the maximal CV at 2%. Therefore, the optimal configuration for 36 LEDs was selected for this application. By comparing the values in Table 5, it can be seen that the more LEDs are used, the higher the average irradiance in the target area for a similar uniformity. The selection of which configuration to choose is now based on the trade-off between price and irradiance. Higher irradiance levels are more desirable because then a smaller exposure time can be used, which can further improve the motion blur and allow a larger depth of field. However, the more LEDs used, the higher the cost of the system. Based on previous experiments it was found that 450 W/m^2^ was necessary, therefore the configuration with 36 LEDs was chosen as the optimal configuration for this application. The LED positions are illustrated in Figure 11 and the irradiance distribution is given in Figure 12. The minimum irradiance in the target area was 415.8 W/m^2^ while the maximal value was 467.1 W/m^2^, resulting in a min to max ratio of almost 90%. It is clear that the algorithm performs better than the traditional approaches using a square or circular array of LEDs in terms of homogeneity. In contrast to the local search algorithm of [17], this algorithm guarantees a symmetrical distribution of LEDs which can be important for design purposes. Furthermore, it is more flexible because it does not only optimize the uniformity of lighting in the target area, but also takes into account the required irradiance level (multiple objective). For thermal design, restrictions were set for the separation between LEDs and for practical purposes the size of the source plane was restricted as well. Although in this approach it was chosen to use the CV to measure the uniformity of distribution, objective functions using other variables or restrictions can be introduced into the algorithm. To prevent ending up in a local optimum, the parameters for the algorithm (degrees of freedom) to optimize, were limited to the x- and y-position of LEDs in one quadrant. In theory it is possible to extend the search space, for example by including a third spatial dimension or by including different types of LEDs, lenses or LED orientations. This however decreases the possibility of finding a global solution and the algorithm could end up in local optima. Furthermore, it is possible that some of these conditions would increase the complexity, sensitivity and cost of the final system.

## 5. Conclusions

In this paper, a LED based illumination system for high speed motion estimation of fertilizer particles was developed. To maximize the accuracy, a high irradiance is necessary without compromising the uniformity of light distribution. In a first step, the optimal LED was selected from a range of commercially available power LEDs by taking into account the camera sensitivity. Next, the optimal configuration of LEDs was determined using a multiple objective genetic algorithm. Both the irradiance and homogeneity of light distribution were considered, in contrast to approaches used in literature. The angular distribution pattern from the manufacturer was used for simulations. Comparing simulation results for three types of lenses, the narrow angle lens was found optimal for this application. Multiple Pareto optimal solutions were simulated for different numbers of LEDs and from this set, the best configuration was selected. The optimal configuration had an irradiance of 452 W/m^2^ and coefficient of variation below 2%. The algorithm proved superior to other approaches in literature can be used and extended for various other applications.
